# A combination of surgery, theranostics, and liquid biopsy - a personalised oncologic approach to treatment of patients with advanced metastatic neuroendocrine neoplasms

**DOI:** 10.7150/ijms.51740

**Published:** 2021-03-19

**Authors:** Andrea Frilling, Ashley K. Clift, Adam E. Frampton, Jamshed Bomanji, Daniel Kaemmerer, Adil Al-Nahhas, Ali Alsafi, Mark Kidd, Irvin M. Modlin, Dieter Hoersch, Richard P. Baum

**Affiliations:** 1 Department of Surgery and Cancer, Imperial College London, London, UK.; 2 Department of Nuclear Medicine, University College London Hospitals, London, UK.; 3 Department of General and Visceral Surgery, Zentralklinik Bad Berka, Bad Berka, Germany.; 4 Department of Imaging and Nuclear Medicine, Imperial College London, London, UK.; 5 WREN Laboratories, Branford, USA.; 6 Gastroenterological and Endoscopic Surgery, Yale University School of Medicine, New Haven, USA.; 7 Department of Gastroenterology/Endocrinology, Zentralklinik Bad Berka, Bad Berka, Germany.; 8 CURANOSTICUM Wiesbaden-Frankfurt at DKD Helios Klinik, Wiesbaden, Germany.

**Keywords:** Neuroendocrine neoplasia, small bowel, surgery, peptide receptor radionuclide therapy, NETest, mRNA, multianalyte gene biomarker.

## Abstract

**Rationale:** Neuroendocrine neoplasia (NEN) of small bowel (SBNEN) frequently present with metastatic disease. Theranostics (molecular imaging followed by targeting therapy) allow for personalised medicine. Liquid biopsies enable precise identification of residual disease and real-time monitoring of therapeutic response. Our aim was to determine the clinical utility of a combination of surgery, theranostics, and a multigene blood measurement in metastasised SBNEN.

**Methods:** Inclusion criteria were SBNEN, G1/G2 NEN, initial tumour diagnosis, stage IV NEN, positivity on ^68^Ga somatostatin analogue PET/CT, eligible for surgery, and ^177^Lu peptide receptor radionuclide therapy (PRRT). Blood samples for NETest were collected longitudinally. Progression-free survival (PFS) and overall survival (OS) were calculated. NETest results were assessed prior to surgery and during clinical follow-up.

**Results:** A surgical cohort of 39 SBNEN patients met eligibility criteria. Thirty-two patients underwent ileal resection and 7 right hemicolectomy. The mean number of ^177^Lu PRRT cycles was 4. Mortality was nil. Surgical morbidity was 10.3%. Transient grade 1/2 toxicity occurred in 41% (PRRT). NETest scores (n=9 patients) decreased in 100% following treatment and correlated with diminished tumour volume and disease stabilization following surgery and PRRT. Median follow-up: 78 months. Median PFS and OS: 42.7 and 110 months, respectively. Progression-free survival at 1-, 3-, and 5-years was 79.4%, 57.1% and 40.5%, respectively. Overall survival at 1-, 3-, and 5-years was 97.4%, 97.4%, and 94.1%, respectively.

**Conclusions:** Surgery combined with ^177^Lu PRRT is safe and provides favourable PFS and OS in selected patients with advanced SBNEN. Liquid biopsy (NETest) has the potential to accurately delineate disease status.

## Introduction

Patients with well-differentiated, grade (G) 1 and G2 entero-pancreatic neuroendocrine neoplasms (NEN) are frequently metastatic at initial diagnosis. Metastases are detectable in 45%-90% of patients with NEN originating from small bowel (SBNEN) 1, 2 and in 40%-80% of pancreatic NEN (PanNEN) 3. First line treatment is provided by radical surgery to eliminate the primary tumour including loco-regional lymph node metastases and liver metastases and provides the best long-term outcome 4, 5.

A substantial number of SBNEN patients and those with NEN originating from the pancreas are diagnosed with resectable primary tumours in the presence of distant metastases not amenable to conventional surgical resection. There is clear indication for resection of the primary tumour in patients who are symptomatic due to functional syndromes and/or loco-regional tumour-related symptoms regardless of the disease stage. However, there is controversy regarding benefit of resection of asymptomatic SBNEN or PanNEN in the presence of unresectable liver metastases 6-8. In the absence of randomised controlled trials, a systematic review showed an improved pooled 5-year overall survival of 73.1% (range 57-81%) for patients with resected SBNEN and unresectable liver metastases compared to 36.6% (range 21%-46%) in the unresected group 9. Recently, a number of effective targeted treatment options have been introduced into the palliative management of patients with metastasised, non-resectable NEN. These include long-acting repeatable (LAR) somatostatin analogues, everolimus, sunitinib, and systemic peptide receptor radionuclide therapy (PRRT) with Yttrium-90 (^90^Y)-DOTA^0^-Try^3^octreotide (^90^Y-DOTATOC) 10 or with Lutetium-177 (^177^Lu)-DOTA^0^-Try^3^-octreotate (^177^Lu-DOTATATE) or ^177^Lu-DOTATOC 11,28. Thus, a promising, novel approach of evolving interest has been the introduction of multimodal therapy combining surgery with patient-tailored systemic therapies targeting non-resectable disease.

'Theranostics' refers to the use of molecular targeting vectors labelled with radionuclides which can be used for both diagnostic imaging and tumour-specific therapy 12, 13. Neuroendocrine neoplasms represent an ideal condition for theranostic approaches as most well- and moderately-differentiated NEN overexpress somatostatin receptors (SSTR) suitable for targeting with radiolabelled peptides for both diagnostic and therapeutic purposes 12, 14. First, the tumour is visualised with ^68^Gallium (^68^Ga) labelled DOTA-peptides with high binding affinity to somatostatin receptors utilising the positron emission (PET) / computed tomography (CT) (PET/CT) 15. Second, DOTA-peptides radiolabelled with a therapeutic radionuclide such as ^177^Lu or ^90^Y are applied for therapeutic purpose. The efficacy of PRRT in terms of favourable progression-free survival (PFS) and overall survival (OS) in NEN patients compared to historical controls has been shown in numerous phase I and phase II studies 16-19, in larger patient cohorts 20, 21 and in the phase III trial of ^177^Lu-DOTATATE in patients with advanced well-differentiated midgut NEN with disease progression during first-line somatostatin analogue therapy (NETTER-1 trial, NCT 01578239) 11. In the ^177^Lu-DOTATATE group, PFS at month 20 was 65.2% (95% confidence interval [CI] , 50.0 to 76.8) compared to 10.8% (95% CI, 3.5 to 23.0) in the control group. A meta-analysis on efficacy of ^177^Lu PRRT in patients with unresectable metastasised NEN demonstrated a pooled disease response rate of 29.1% (95% CI: 20.2%-38.9%), and disease control rate of 74.1% (95% CI: 67.8%-80.0%) 22.

Liquid biopsies have garnered rising interest over the last few years given their potential for longitudinal, real-time status assessment and provision of diagnostic, prognostic, and predictive markers for precision medicine 23, 24. A blood-based 51-gene multianalyte transcript biomarker has been developed specifically for well-differentiated NEN to dynamically characterise tumour behaviour from genomic information 25. The test (NETest) is >90% accurate in NEN originating from the gastro-entero-pancreatic and bronchopulmonary system 26-28. Its main clinical aim is to monitor disease progress, predict therapeutic efficacy and assess prognosis. In addition, it can be used to delineate the effectiveness of operative resection and ablation strategies and identify minimal residual disease 29, 30. In direct comparative studies it outperforms the current standard tumour marker chromogranin 25. The sensitivity and specificity of the NETest is >95% and >90%, respectively 31. In a US Registry-based study, NETest diagnostic accuracy was 96% and concordant with image-demonstrable disease in 95% (NCT 02270567) 28. High NETest score correlated with progressive disease and low NETest score with stable disease.

In this study, we aimed to evaluate the outcome of a multimodal approach combining surgery and theranostics in well-characterised group with metastatic small bowel neuroendocrine neoplasms. In addition, we investigated whether a multianalyte gene transcript biomarker could assess disease status and delineate disease reduction following multimodal treatment.

## Patients and Methods

Consecutive patients with small bowel NEN (SBNEN) treated between 01 January 2007 and 31 December 2018 at Imperial College Health Care NHS Trust (ICHCNT), London, UK and at the Zentralklinik Bad Berka (ZKBB), Bad Berka, Germany, respectively were considered in this longitudinal study. Both institutions, which are Centres of Excellence of the European Neuroendocrine Tumour Society, followed the same diagnostic and treatment protocols. All patients were discussed in local multidisciplinary team meetings. Clinical information was prospectively collected in institutional NEN databases.

**Figure 1:**
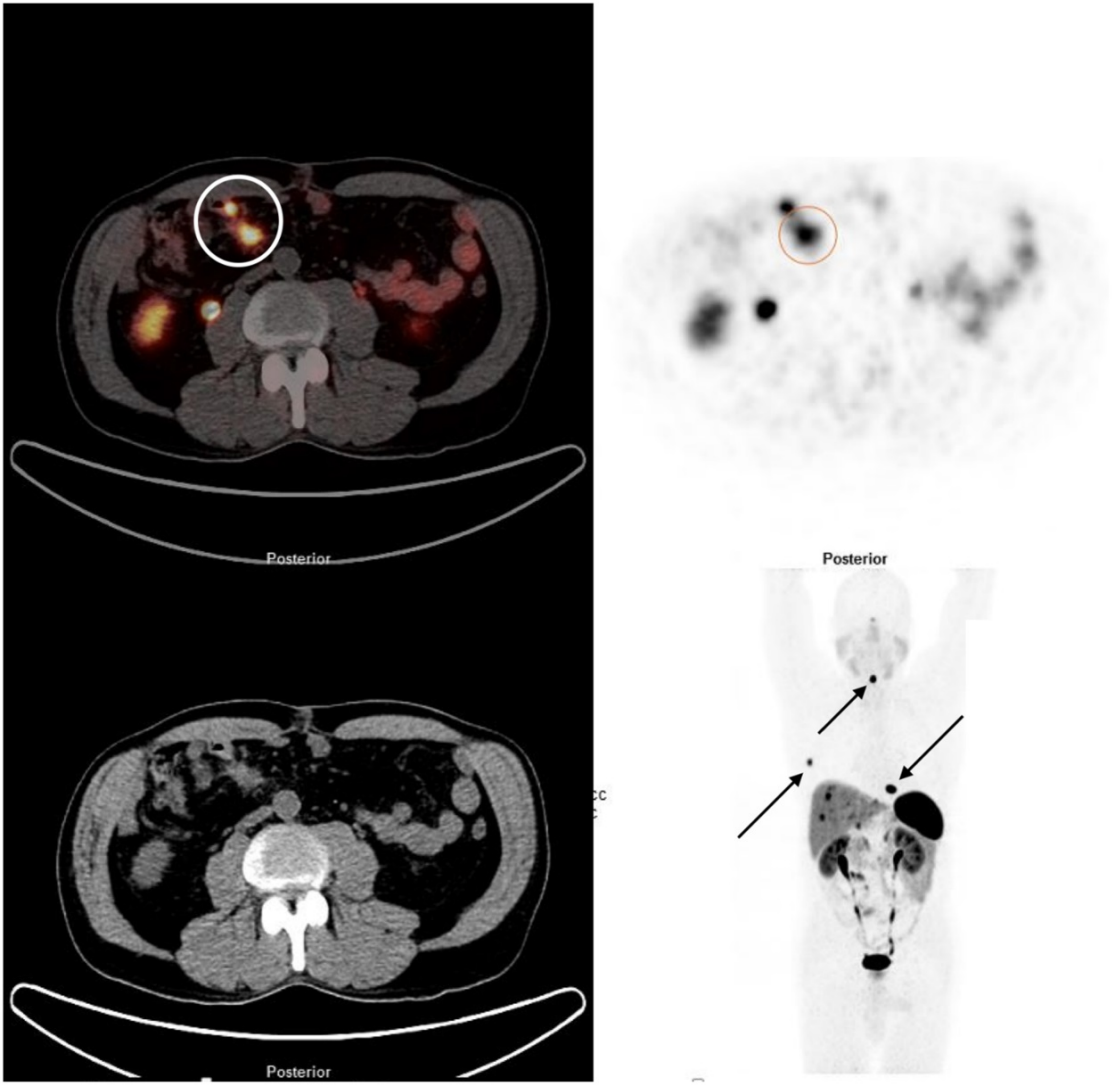
^68^Ga DOTATATE PET/CT demonstrating a SBNEN with lymph node metastases and distant metastases. The primary tumour and locoregional lymph node metastases (stage I) are marked with a circle on the fused image (left side) and on the PET component (right side). The patient also had multiple bone metastases (rib, cervical spine and thoracic spine [arrows]) and liver metastases.

Inclusion criteria were well- or moderately differentiated, grade (G) 1 and G2 NEN originating from small intestine, no previous treatment for NEN apart from injections of high-dose octreotide LAR for amelioration of hormonal symptoms associated with carcinoid syndrome, symptoms caused by loco-regional tumour growth, unresectable stage IV disease, positivity on ^68^Ga-DOTA-D-Phe1-Tyr3-octreotide (DOTATOC) PET/CT, ^68^Ga-DOTA-1-Nal3-octreotide (DOTANOC) PET/CT, and ^68^Ga- DOTA-D-Phe1-Tyr3-Thr8-octreotide (DOTATATE) PET/CT, respectively (Figure 1), absence of severe carcinoid heart disease, eligibility for surgery within four weeks following initial diagnosis, and eligibility for ^177^Lu PRRT within three months following surgery. Patients who had symptoms of carcinoid syndrome and were referred to us having not yet started treatment for such symptoms were started on octreotide LAR. The treatment was discontinued prior to PRRT with a withdrawal period of 4-6 weeks. Inclusion of patients at ICHCNT was impacted by limited availability of PRRT in UK during the study period. Resectability of liver metastases was assessed with MRI and SSTR-PET/CT. Follow-up encompassed standard biochemistry, morphologic imaging every 3-6 months, SSTR-PET/CT based imaging every 6-12 months, and in a subgroup of patients, blood sampling for NETest prior to surgery and at 6 months after the last PRRT cycle. Overall efficacy of treatment and response to PRRT were assessed with SSTR-PET/CT at 6 months after the last treatment cycle. Outcome measures included 1-, 3-, and 5-year progression-free (PFS) and overall survival (OS) from initial diagnosis. Only patients with a follow-up of at least 6 months were considered.

SSTR-PET/CT was performed according to standard protocols as described previously 32, 33. The criteria used to define eligibility for PRRT were in accordance with published guidelines for PRRT and included a Karnofsky index of >60%, life expectancy of more than 6 months, somatostatin receptor positive NEN and adequate bone and renal function 33. In short, 7.4 GBq (200 mCi) of ^177^Lu-DOTATATE (Lutathera®) (ICHCNT) and ^177^Lu-DOTATOC with a mean applied activity of 6.54 GBq (ZKBB) was infused intravenously over a period of 15-30 minutes. Most patients received three to four infusions every eight to 12 weeks. An intravenous reno-protective amino acid solution was administered prior to the administration of the therapeutic dose and continued for four hours thereafter 33. Individual therapy planning based on pre-therapy SSTR-PET/CT as the dosimetry protocol and SPECT/CT studies acquired at 24h after each PRRT cycle were considered. With individual dosimetry we aimed to establish personalised treatment doses to ensure that the total renal dose and the bone marrow dose did not exceed the 23 Gy and the 2 Gy limit, respectively.

Regular blood tests for assessment of adverse events and imaging were carried out. Final restaging and assessment of response to treatment were performed with SSTR-PET/CT at 6 months after the last PRRT cycle. Responses were evaluated with European Organisation for Research and Therapy of Cancer (EORTC) criteria 34 (PET component of PET/CT) as well as by Response Evaluation Criteria in Solid Tumors (RECIST) 35 (CT component of PET/CT or MRI). Adverse events were assessed from laboratory data at the time of occurrence and graded according to the Common Terminology Criteria for Adverse Events (CTCAE) version 4.0 (NCI, Bethesda, USA).

Our standard surgical approach included systematic palpation of the entire small bowel starting at the Treitz ligament and ending at the caecal valve, assessment of mesenteric lymph node metastases involvement (stage I-IV) 36, and segmental small bowel resection/s including mesenteric loco-regional disease. A lymph node-first, intestinal-sparing principle was followed. For tumours localized in the terminal ileum, concomitant oncologic right hemicolectomy was performed. All patients with liver metastases and /or carcinoid syndrome were treated with octreotide (50 microgram/hour intravenously) for 12 hours prior to surgery and 24h thereafter. Resected specimens were subjected to immuno-histochemical examination including assessment of Ki67% for tumour grading. Surgical morbidity was assessed according to Clavien-Dindo classification 37 and mortality recorded.

### Multigene blood analysis

Only patients seen at ICHCNT and treated between 2014 and 2018 were considered as NETest candidates for logistic reasons. In a sub-cohort of patients (SBNEN, n=9), blood samples for the NETest were collected prior to surgery and at 6 months following the last PRRT cycle. All patients considered for NETest completed blood sampling according to the protocol. The analysis comprised a 2-step protocol (RNA isolation/cDNA production and qPCR) from EDTA-collected whole blood 25. Transcripts (mRNA) were isolated from EDTA-collected whole blood samples (mini blood kit, Qiagen, Valencia CA) and real-time PCR performed on pre-spotted plates. Target transcript levels were normalized and quantified versus a population control. Results were expressed as an activity index (NETest score) from 0-100 ('normal' score range is 0-20). Assays were undertaken using de-identified samples in a central USA clinically and federally certified laboratory (Wren Laboratories CL-0704, CLIA 07D2081388). NETest results were correlated with clinical follow-up data. The study was approved by the Imperial College Healthcare Tissue Bank Committee and a National Research Ethics Committee (07/MRE09/54). Informed consent was obtained from all patients.

### Statistical analysis

Patient demographics and tumour characteristics were reported with descriptive statistics. Progression-free survival and OS were recorded by using Kaplan-Meier methodology and were calculated from initial diagnosis to the date of disease progression evident on imaging and the date of death or last-follow up visit, respectively. Mann-Whitney U-test was used to evaluate changes in NETest. A *p* value of <0.05 was deemed statistically significant. Stata v15 was used for statistical analyses.

## Results

### Surgery

**Figure 2:**
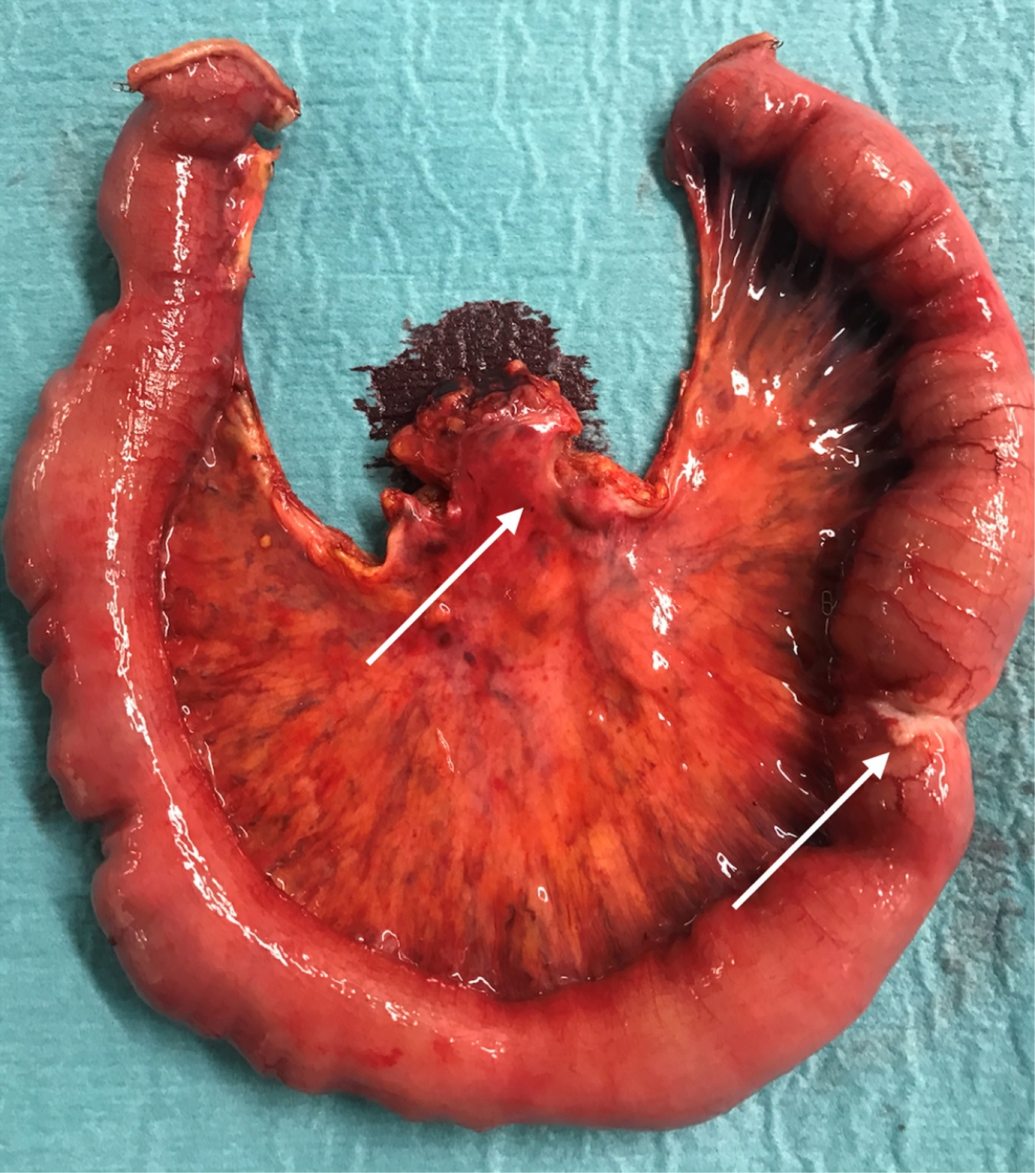
Surgical specimen of a patient with a SBNEN (arrow) causing intermittent bleeding and bowel occlusion. In addition, 11 further tumours all sized ≤5 mm and only detectable on palpation were found and confirmed as NEN on immunohistological examination. There were stage III mesenteric lymph node metastases (arrow).

Thirty-nine SBNEN patients (24 males (61.5%) median age at surgery 58.8 years [range 32.1-78.4]) were enrolled prospectively (Table 1). During the study period, 395 SBNEN patients were treated in total. All patients were symptomatic due to local mass effect and/or hormonal excess. In all patients, anatomic imaging and/or SSTR-PET/CT revealed distant metastases not suitable for resection. Of the SBNEN patients, 32 (82.1%) underwent ileal resection(s) and mesenteric lymphadenectomy and in 7 (17.9%), concomitant oncologic right hemicolectomy was carried out. Twelve patients (30.8%) had multifocal tumours (mT1-T3). The majority of patients with unifocal primary tumours had T2 and T3 lesions, respectively (88.9%). Mesenteric lymph node metastases were evident in 37/39 patients (94.9%) and resected in 35/37 (94.6%) (Figure 2). In two patients with stage IV lymph node metastases and liver metastases, resection of the metastatic lymph node bulk was abandoned to avoid the risk of bowel ischemia and short bowel syndrome. In all patients, surgery took place within 4 weeks following initial diagnosis. All 35 patients with liver metastases (89.7%) had type II (isolated metastatic bulk accompanied by smaller deposits), or type III (disseminated metastatic spread) liver metastases with >75% liver parenchyma having evidence of metastatic involvement. Even under consideration of the ≤70% rule for debulking of NEN liver metastases, down-staging procedures, and two-stage hepatectomies, they were considered as non-resectable.

The 90-days postoperative morbidity was 10.3% (4/39) (all grade 1). The 90-days postoperative mortality was nil. The symptoms caused by loco-regional tumour extent recorded preoperatively diminished in all cases after surgery. Histology confirmed well-differentiated NEN grade 1 or grade 2 in all resected specimens.

### ^177^Lu PRRT

Treatment was commenced in all 39 patients within 3 months following surgery. The mean number of ^177^Lu-octreotate cycles was 4 (range 2-6). The administered activity dose and the number of treatment cycles were determined with individual dosimetry. Transient grade 1/2 toxicity occurred in 16 patients (41%). There was no grade 3 toxicity. At 6 months following the last cycle, complete response, partial response and stable disease were seen in 3 (7.69%), 25 (64.1%), and 11 (28.2%) patients, respectively. In the two patients with remaining stage IV lymph node metastases (both also had liver metastases), complete response and stable disease, respectively were achieved. No disease progression was recorded.

### Multigene blood analysis

**Figure 3:**
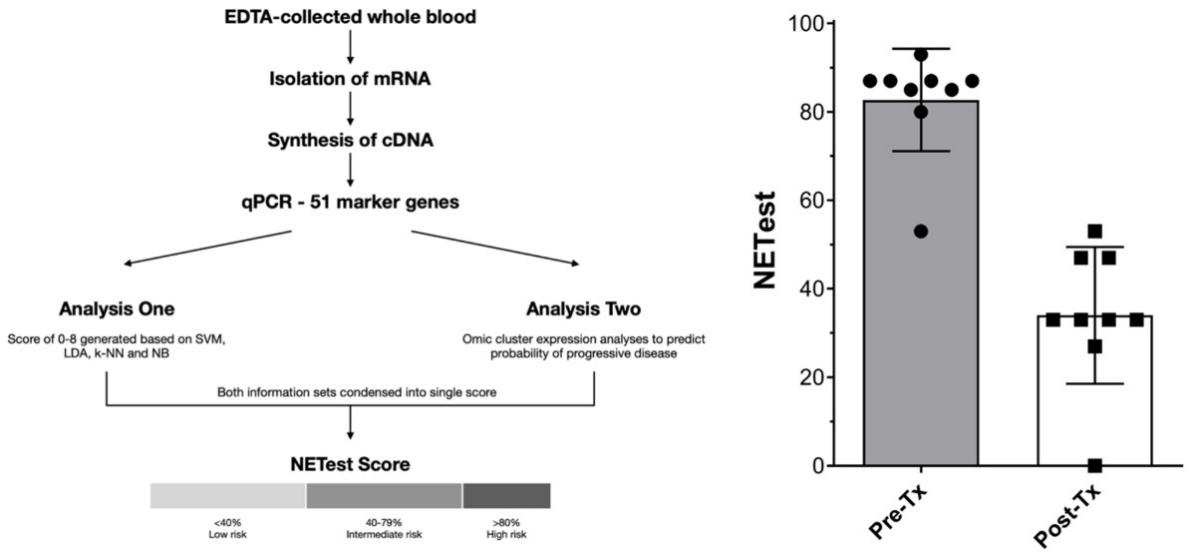
Representation of steps involved in NETest assay and derivation of results (3A, left) and NETest results pre-treatment and post-treatment (surgery followed by PRRT) for 9 patients in this study (3B, left).

Nine patients were available for inclusion. The NETest was positive in 9/9 (100%) patients prior to treatment and 8/9 scores were ≥80 (consistent with advanced disease, Figure 3). After treatment (surgery and PRRT), NETest scores dropped from 83±12 to 34±15 (*p*<0.0001) (Figure 3) concordant with imaging results in all 9 patients. Six exhibited scores ≤40 (consistent with stable disease), 3 exhibited scores of 47-53. These scores preceded further therapy and disease status remained unchanged since the post-treatment NETest in all during further follow-up (median 18.5 months, range 6 to 46months).

### Survival outcomes

**Figure 4:**
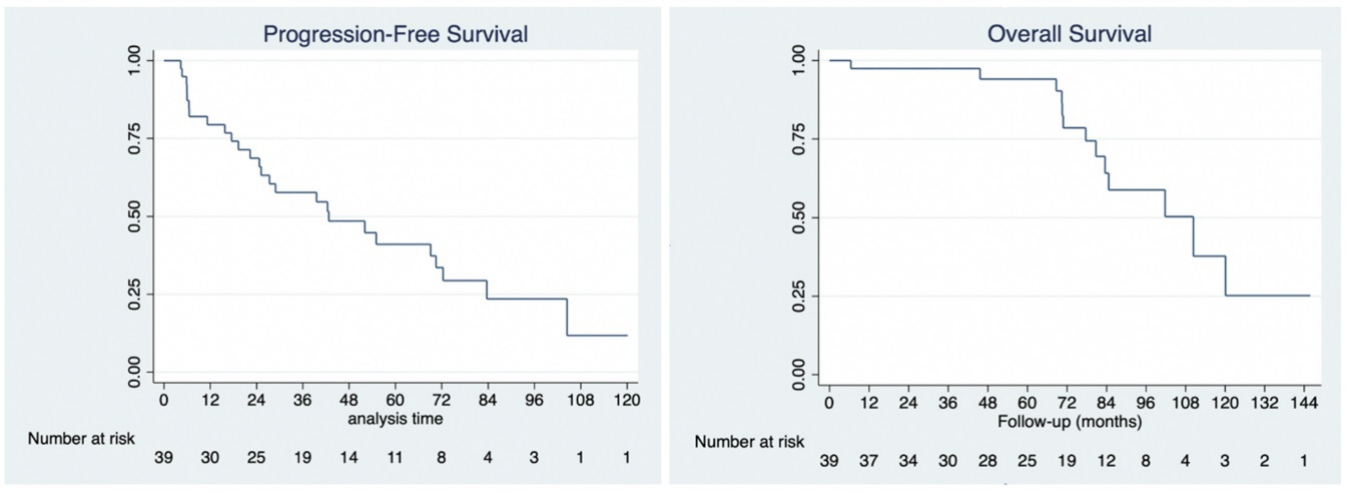
Kaplan-Meier analysis of progression-free survival (4A, left) and overall survival (4B, right).

No patient was lost to follow-up. The median follow-up for the cohort was 78 months (range: 6 to 146 months), during which there were 13 deaths, all due to disease. Of all 39 patients, 26 developed progressive disease and required further treatment (not reported here). Median overall survival was 110 months (95% CI: 80.8 to 138.7 months). Overall survival at 1-year, 3-years and 5-years was 97.4%, 97.4% and 94.1%, respectively. Median progression-free survival was 42.7 months (95% CI: 24.7 to 72.4 months). Progression-free survival at 1-year, 3-years and 5-years was 79.4%, 57.1%, and 40.5%, respectively (Figure 4).

## Discussion

Small bowel NEN frequently present with synchronous distant metastases, undermining the efficacy of surgical treatment. Widespread use of SSTR-based PET/CT technology has improved detection of metastatic deposits and predicates upstaging of 30%-40% of NEN patients with only localised disease seen on standard imaging and change in treatment strategy in up to 45% by identifying lesions that are suitable for resection or detecting multiple unresectable lesions approachable by non-surgical therapeutic modalities 38-40. Particularly challenging clinical scenarios include patients with NEN that are symptomatic due to local tumour effect since they require both surgery to eliminate the primary tumour and systemic treatment for metastases.

Thyroidectomy followed by ^131^Iodine (^131^I) radiotherapy for treatment of differentiated thyroid cancer accounts for an *example par excellence* of a multimodal treatment concept combining surgery for the primary tumour and theranostics for management of its metastatic and/or residual disease 41. In NEN, combining surgery and targeted molecular radionuclide therapy (PRRT) has been applied mainly in neoadjuvant settings for down-staging of resectable or potentially resectable tumours 42-44, or as an upfront strategy to enhance response to PRRT and prolong survival 43, 45-47.

Our treatment strategy consisted of three steps; accurate staging with molecular imaging, surgery for elimination of loco-regional disease, and molecular therapy for targeting of unresectable metastases. With a median PFS of 42.73 months and OS at 5-years of 94.1% we have demonstrated in a group of stage IV SBNEN patients the efficacy of this approach. Surgical morbidity (all grade 1) of 10.3%, and no relevant toxicity (all grade 1/2) of PRRT further underlined the justification of our concept. An overall response rate to PRRT of 71.8% achieved in this study confirmed favourable observations made by others treating specifically advanced midgut NEN with PRRT 11, 20. We speculate that the favourable results achieved in our study may be related to manifold factors. Our patient cohort was very homogeneous (G1/G2 SBNEN only), our patients were relatively young (the median patient age was <60 years) and all patients underwent surgical treatment within 4 weeks after initial diagnosis, of which >90% had radical resection of locoregional disease. Lastly, PRRT utilising personalised dosimetry was applied within 4 months of initial diagnosis, and was first-line therapy for non-resectable disease prior to progression in all patients. Because of the limited efficacy of somatostatin analogues in patients with SBNEN and high hepatic tumour burden 48, the high success rate of therapy with 177Lu-octreotate when compared to somatostatin analogues 11 and the absence of serious side-effects, we and others 49 advocate its use in patients with SBNEN without waiting for tumour progression. In addition, it has been shown that the effect of PRRT negatively correlates with the volume of hepatic metastatic tumour burden.

Surgery of SBNEN might be challenging due to two characteristic features of these tumours; primary tumour multifocality in up to 30% and mesenteric lymph node metastases frequently compromising major mesenteric vascularity (stage III) or encasing the mesenteric root (stage IV). Radical surgery has been shown to reduce the risk of local complications and increase long-term survival 1. In this series, multifocal tumours were present in 30.8% and >95% had mesenteric lymph node metastases of various stages. Although laparoscopic resection of SBNEN has been reported 50,51, we are in favour of laparotomy allowing for meticulous palpation of the entire small bowel and identification of frequently millimetre-sized intramural tumours not seen preoperatively on any imaging or endoscopy 52, and optimal extent of lymphadenectomy (at least 8 lymph nodes) under consideration of intestinal-sparing principles 53, 54. Hand-held gamma probe surgery using gallium-68-labeled somatostatin analogues has been shown to be a promising adjunct for real-time detection of small metastatic deposits and primary tumours 55, 56.

Personalized or precision medicine utilising high-throughput omics technologies including genomics, transcriptomics, metabolomics, proteomics, and epigenomics has gained increasing attention in the management of oncologic patients 57. Recently, Sausen *et al.* have shown in patients with pancreatic adenocarcinoma with localized disease on imaging at initial diagnosis that circulating tumour DNA (ctDNA) was detected in 43% of cases, which predicted recurrence, poorer overall survival, and detected disease recurrence 6.5 months earlier than standard imaging 58.

The multianalyte gene transcript biomarker was developed for well-differentiated NEN and in our study provided information consistent with observations regarding clinical usefulness of liquid biopsies in other malignancies 23. NETest was informative regarding the clinical diagnosis of a NEN, delineation of tumour burden reduction, response to treatment, and reflected disease status at follow-up. Filosso *et al.* demonstrated the clinical utility of NETest in a surgical series of patients with various types of lung malignancies, of them 28 with typical and atypical carcinoids, respectively. NETest accurately identified carcinoids, differentiated progressive from stable disease and verified completeness of surgical resection 30. Genc *et al.* have shown in well-differentiated PanNEN that combining standard clinico-pathologic criteria and NETest results were approximately twice as effective as individual clinico-pathologic criteria alone since it positively correlated with recurrence in R0 resected patients 59. A recent meta-analysis of NETest data by Oberg, et al. including ten original publications confirmed the diagnostic accuracy to be 95-96% and that chromogranin A which accounts for the standard tumour marker for NEN had no clinical utility 60. In our own study, the NETest confirmed tumour mass reduction and could be used to stage patients post-surgery. As all had low-intermediate scores, there was no requirement for further therapeutic interventions. The NETest also functioned as an effective surrogate marker for stable disease and may have value in reducing imaging as has been noted 28.

Our study has some limitations. First, we have considered a strongly selected group of patients of whom all presented at initial diagnosis and were treatment naïve. All underwent surgery within 4 weeks after diagnostic completion and PRRT within 3 months following surgery, which does not necessarily reflect a real-world experience. In an ideal environment, cancer patients should be offered treatment within shortest time. Regretfully this is very frequently not the case in NEN patients. A recent survey showed that 50% of US patients reported being diagnosed with other conditions before receiving their NEN diagnosis which for 34% took 5 years or more and that there was delay in treatment after the diagnosis 61. Similar experience has been reported in other countries 62. Even when a NEN is assumed, it can take substantial time to complete the biochemical diagnostic process and secure a timely slot for somatostatin receptor-based PET/CT imaging. Starting with PRRT within 3 months following surgery may also be challenging due to: A) postoperative morbidity (e.g. anastomotic leakage or delayed /incomplete wound healing) precluding timely PRRT, B) not all centres have sufficient capacity to offer PRRT to a large number of patients [limitation of treatment sessions/week due to radiation protection regulations], C) depending upon local structures, shortage/delay in drug delivery may occur, and D) unforeseen technical issues may lead to *ad hoc* treatment session cancellation.

Second, we had no control group which would allow precise assessment of clinical significance of the concept presented in our study. Only a large, comparative treatment study would be able to determine “efficiency” of a modality in real word setting. This is unlikely to happen in NEN.

Third, although our data and experience of other groups suggest that NETest seems to be useful in the assessment of the adequacy of a treatment modality 31, 63, 64, a further long-term prospective study including a large number of consecutive patients is needed to establish the most accurate timing of post-interventional blood sampling as well as the metrics of the NETest in the prediction of residual or recurrent disease.

In conclusion, combining molecular imaging, surgery, and targeted molecular therapy provides a promising and safe approach for treatment of patients with well-differentiated metastasised SBNEN. The multianalyte biomarker NETest seems to be a promising tool for detection of neuroendocrine disease. It has the potential to define completeness of treatment and disease status. It may be used as a marker for disease stabilization. The efficacy of our approach provides a basis to support current efforts to establish the further development of precision treatment of oncologic patients.

## Figures and Tables

**Table 1:** Clinical and pathological characteristics of the study cohort (n=39).

Characteristic	Number of patients (%)
**Sex**	
Male	24 (61.5)
Female	15 (38.5)
Age, median (range)	58.8 years (32 to 78 years)
**Primary tumour site**	
Small bowel	39 (100)
**Tumor focality**	
Unifocal	27 (69.2)
Multifocal	12 (30.7)
**Symptoms due to mass effect**	
Pain	39 (100)
Bleeding	15 (38.5)
Intestinal obstruction	6 (15.4)
**Functional symptoms**	
Carcinoid syndrome	25 (64.1)
**Surgical procedures (small bowel)**	
Segmental resection/s	32 (82.1)
Right hemicolectomy	7 (17.9)
**Tumour grade**	
Grade 1	30 (76.9)
Grade 2	9 (23.1)
Grade 3	0 (0)
**Tumour stage**	
T1	0 (0)
T2	14 (35.9)
T3	10 (25.6)
T4	3 (7.7)
mT1-T3	12 (30.8)
Lymph node metastases	37 (94.9)
**Distant metastases**	
Liver	35 (89.7)
Bone	16 (41)
Peritoneum	4 (10.3)
Other	2 (5.1)

mT=multifocal tumors.
